# miR-142-3p Regulates BDNF Expression in Activated Rodent Microglia Through Its Target CAMK2A

**DOI:** 10.3389/fncel.2020.00132

**Published:** 2020-05-21

**Authors:** Neelima Gupta, Shweta Jadhav, Kai-Leng Tan, Genevieve Saw, Karthik Babu Mallilankaraman, S. Thameem Dheen

**Affiliations:** ^1^Department of Anatomy, Yong Loo Lin School of Medicine, National University of Singapore, Singapore, Singapore; ^2^Department of Physiology, Yong Loo Lin School of Medicine, National University of Singapore, Singapore, Singapore

**Keywords:** rodent primary microglia, microglia BV2, microRNA, microarray, microRNA-142 (miR-142-3p/5p), Ca^2+^/calmodulin dependent kinase 2a (CAMK2A), brain-derived neurotrophic factor (BDNF)

## Abstract

Microglia, the innate immune effector cells of the mammalian central nervous system (CNS), are involved in the development, homeostasis, and pathology of CNS. Microglia become activated in response to various insults and injuries and protect the CNS by phagocytosing the invading pathogens, dead neurons, and other cellular debris. Recent studies have demonstrated that the epigenetic mechanisms ensure the coordinated regulation of genes involved in microglial activation. In this study, we performed a microRNA (miRNA) microarray in activated primary microglia derived from rat pup’s brain and identified differentially expressed miRNAs targeting key genes involved in cell survival, apoptosis, and inflammatory responses. Interestingly, miR-142-3p, one of the highly up-regulated miRNAs in microglia upon lipopolysaccharide (LPS)-mediated activation, compared to untreated primary microglia cells was predicted to target Ca^2+^/calmodulin dependent kinase 2a (CAMK2A). Further, luciferase reporter assay confirmed that miR-142-3p targets the 3′UTR of *Camk2a*. CAMK2A has been implicated in regulating the expression of brain-derived neurotrophic factor (BDNF) and long-term potentiation (LTP), a cellular mechanism underlying memory and learning. Given this, this study further focused on understanding the miR-142-3p mediated regulation of the CAMK2A-BDNF pathway *via* Cyclic AMP-responsive element-binding protein (CREB) in activated microglia. The results revealed that CAMK2A was downregulated in activated microglia, suggesting an inverse relationship between miR-142-3p and *Camk2a* in activated microglia. Overexpression of miR-142-3p in microglia was found to decrease the expression of CAMK2A and subsequently BDNF through regulation of CREB phosphorylation. Functional analysis through shRNA-mediated stable knockdown of CAMK2A in microglia confirmed that the regulation of BDNF by miR-142-3p is *via* CAMK2A. Overall, this study provides a database of differentially expressed miRNAs in activated primary microglia and reveals that microglial miR-142-3p regulates the CAMK2A-CREB-BDNF pathway which is involved in synaptic plasticity.

## Introduction

Microglia, the resident macrophages of the central nervous system (CNS), not only serve as the first line of defense in the brain but also contributes to the maintenance of brain homeostasis (Li and Barres, 2018). They become activated in response to various stimuli in CNS pathologies such as brain injury, neurodegenerative diseases, and infection (Zielasek and Hartung, 1996). The activated microglia appear morphologically hypertrophic, migrate to the site of injury and release several proinflammatory cytokines, chemokines, cytotoxic molecules such as nitric oxide and reactive oxygen species (Chao et al., 1992; Banati et al., 1993; Fetler and Amigorena, 2005; Thameem Dheen et al., 2007). In addition to their immune functions, microglial cells play an important role in sculpting the neuronal circuits during brain development (Wu et al., 2015). In a developing brain, neurons form an extensive network of synaptic connections, which are eventually pruned by microglial cells to establish a mature neuronal network (Schafer and Stevens, 2013; Schafer et al., 2013; Sierra et al., 2013). The activated microglial cells have been shown to remove damaged or dysfunctional synapses from injured neurons in a process called synaptic stripping (Kettenmann et al., 2013). Many signaling pathways are important for microglia-mediated synaptic maintenance in the healthy and pathological brain. Of note, a recent study reported the significant contribution of microglial brain-derived neurotrophic factor (BDNF) towards learning-dependent synapse formation (Parkhurst et al., 2013; Saw et al., 2020). However, the molecular and epigenetic mechanisms underlying microglia-mediated maintenance of neuronal synapses under health and diseases remain unclear.

An important epigenetic regulator of microglial functions are miRNAs (Karthikeyan et al., 2016) which are small non-coding RNA molecules that influence gene function by targeting the 3′UTR of mRNAs, thus leading to either mRNA degradation or inhibition of protein translation (Mattick, 2009; Taft et al., 2010). MicroRNAs regulating diverse cellular processes and pathways provide a new and potential candidate for therapeutic intervention against various pathological conditions (Gupta et al., 2018; Karthikeyan et al., 2018; Wang and Wang, 2018; Guo et al., 2019). Recent studies have shown miRNAs to be involved in microglial activation in various CNS pathologies such as HIV-1 infection (Mishra et al., 2012; Jadhav et al., 2014; Wallet et al., 2019), Japanese encephalitis virus infection (Thounaojam et al., 2014; Ashraf et al., 2016; Rastogi et al., 2018), Alzheimer’s disease (Guedes et al., 2013; Zhao et al., 2017), ischemia (Zhao et al., 2013; Ni et al., 2015; Li et al., 2019), prion disease (Saba et al., 2012; Slota and Booth, 2019) and signaling pathways such as MAPK signaling (Jadhav et al., 2014; Saika et al., 2017), thus modulating neuroinflammation. Despite recent studies evaluating the role of miRNAs in microglial activation in CNS pathologies, there are no comprehensive profiling studies of miRNA expression patterns in activated microglia. Thus, a disease-specific miRNA profile can be used for diagnosis, prognosis, and treatment of various neuroinflammatory conditions.

Because of the pivotal role of microglial neurotrophins (such as BDNF), an insight into the miRNA-mediated regulation of neurotrophic factors may provide promising biomarkers for various brain diseases. Microglial BDNF is critical for neuron-microglia crosstalk, where BDNF acts on the receptor tyrosine kinase (TrkB) present on dendritic spines involved in synaptic plasticity (Minichiello, 2009).

In the current study, we demonstrate that activation of microglia by lipopolysaccharide (LPS) is associated with the altered miRNA expression profile. This high throughput screening identified several differentially expressed miRNAs in activated microglia. Pathway analysis using the Ingenuity Pathway Analysis (IPA) software identified several signaling pathways to be altered in activated microglia.

For further validation, a highly upregulated microRNA, miR-142-3p in activated microglia was found to target *Camk2a*, a key regulator of microglial BDNF. CAMK2 protein, a member of Ca^2+^/calmodulin (CaM)-dependent protein kinase family, mediates the Ca^2+^-induced signaling which eventually phosphorylates a wide array of substrates to mediate the regulation of cellular responses (Swulius and Waxham, 2008). CAMK2 comprises four very closely related isoforms (a, b, d, and g) encoded by different genes (Hudmon and Schulman, 2002). The CAMK2a and CAMK2b isoforms are highly expressed in the brain, constituting approximately 1% of the total brain protein whereas, CAMK2c and CAMK2d isoforms are expressed throughout the body in very low concentrations (Coultrap and Bayer, 2012; Cook et al., 2018). BDNF, a member of neurotrophic factors, is known to play a critical role in several brain functions such as neuronal development, synaptic plasticity, and learning and memory (Tao et al., 1998; Malenka and Bear, 2004). BDNF transcription is dependent on Ca^2+^ mediated activation of cAMP-response element-binding protein (CREB; Tao et al., 1998). Ca^2+^ acts by activating CAMKs which trigger phosphorylation of CREB (pCREB), which in turn activates BDNF transcription by binding to a cAMP-response element within the gene (Su et al., 2016; Yan et al., 2016). In this study, we focused on understanding the role of microRNA-mediated regulation of CAMK2A-CREB-BDNF signaling pathway in activated microglia.

## Materials and Methods

### Animals

Wistar rat pups (3–5 days old) were purchased from Laboratory Animals Centre, National University of Singapore. This study was approved by the National University of Singapore Institutional Animal Care and Use Committee (IACUC protocol no. R14-1643). All procedures were following IACUC guidelines. All efforts were made to minimize pain and the number of animals used.

### Primary Culture of Microglial Cells

Mixed glial cells were isolated from the cerebrum of 3–5 day postnatal rats and cultured in a flask containing Dulbecco’s modified Eagle’s medium (Cat. No. 1152, DMEM, Sigma, St. Louis, MO, USA), 10% fetal bovine serum (FBS, HyClone, Logan, UT), 10 ml/L antibiotic-anti-mycotic (Cat. No. A5955, Sigma, USA), 0.1 mM nonessential amino acid (Cat. No. 11140-050, Invitrogen, USA) and 1 ml/L insulin (Cat. No. I-0516, Sigma, USA). The complete medium was replaced at 24 h and then every 2–3 days. Microglial cells were isolated at 10 days with 0.25% trypsin containing 1mM Ethylene diamine tetraacetic acid (EDTA) for 15–20 min at 37°C with 5% CO_2_. With a complete detachment of the upper cell layer, microglial cells remained attached to the bottom of the flask and were cultured in complete medium overnight. The purity of microglial cells was confirmed by CD11b labeling (Supplementary Figure S3). The cells were treated with LPS (Cat No. L6529, Sigma-Aldrich) at 1 μg/ml for 6 h to activate microglia.

### BV2 Microglial Cell Culture and Activation

Murine BV2 microglial cells were maintained in Dulbecco’s Modified Eagle’s Medium (DMEM, Cat No. D1152, Sigma-Aldrich) supplemented with 10% fetal bovine serum (FBS, Cat No. SV30160.03, HyClone) and cultured at 37°C with 5% CO_2_ (Blasi et al., 1990). The cells were treated with LPS (Cat No. L6529, Sigma-Aldrich) at 1 μg/ml for 6 h to activate microglia.

### RNA Isolation

Total RNA including miRNAs and small RNAs were extracted from BV2 microglial cells using the miRNeasy Mini kit (Cat No 217004, Qiagen) according to manufacturer’s instructions. RNA isolated was quantified using the Nanodrop spectrophotometer (Thermo Fisher Scientific, Wilmington, DE, USA) and the quality was assessed using a Bioanalyzer (Agilent Technologies Inc., Santa Clara, CA, USA).

### miRNA Profiling

All experiments were conducted at Exiqon Services, Denmark. The quality of the total RNA was verified by an Agilent 2100 Bioanalyzer profile. 400 ng of total RNA from sample and reference was labeled with Hy3^™^ and Hy5^TM^ fluorescent label, respectively, using the miRCURY LNA^TM^ microRNA Hi-Power Labeling Kit, Hy3^TM^/Hy5^TM^ (Exiqon, Denmark) following the procedure described by the manufacturer. The Hy3^TM^-labeled samples and Hy5^TM^-labeled reference RNA samples were mixed pair-wise and hybridized to the miRCURY LNA^TM^ microRNA Array 7th gen (Exiqon, Denmark), which contains capture probes targeting all miRNAs for human, mouse or rat registered in the miRBASE 18.0. The hybridization was performed according to the miRCURY LNA^TM^ microRNA Array instruction manual using a Tecan HS4800^TM^ hybridization station (Tecan, Austria). After hybridization, the microarray slides were scanned and stored in an ozone-free environment (ozone level below 2.0 ppb) to prevent potential bleaching of the fluorescent dyes. The miRCURY LNA^TM^ microRNA array slides were scanned using the Agilent G2565BA Microarray Scanner System (Agilent Technologies Inc., USA) and the image analysis was carried out using the ImaGene 9.0 software (miRCURY LNA^TM^ microRNA Array Analysis Software, Exiqon, Denmark). The quantified signals were background corrected (Normexp with offset value 10, Ritchie et al., 2007) and normalized using the global Lowess (Locally Weighted Scatterplot Smoothing) regression algorithm. For the expression analysis, the *p*-values were calculated based on moderated t-statistics. Furthermore, False Discovery Rates (FDRs) were computed from *p-values* using the Benjamini and Hochberg multiple testing adjustment method. All analyses were conducted in the software R/Bioconductor using the Limma package and an adjusted *p*-value less than 0.05 was used as the stringent cut-off criteria.

### miRNA Real-Time RT-PCR

cDNA conversion for miRNA quantification was performed using the Universal cDNA Synthesis Kit (Prod No. 203301, Exiqon) according to the manufacturer’s instructions. For miRNA quantification, the miRCURY LNA^™^ Universal RT microRNA PCR system (Prod No. 203400, Exiqon) was used in combination with pre-designed primers and snRNA U6 (reference gene). The miRNA expression was quantified using real-time RT PCR system (Model No.7900HT, Applied Biosystems).

### mRNA Real-Time RT-PCR

For mRNA analysis cDNA conversion was carried using 2 μg of RNA, 2 μl of Oligo (dT) 15 primer (Cat. No. C1101, Promega), 1 μl of M-MLV reverse transcriptase (Cat. No. M1701, Promega), 5 μl of M-MLV RT 5× buffer (Cat. No. M531A, Promega), 0.2 μl of RNasin (Cat. No. N2515, Promega), 0.5 μl of dNTP mix (Cat. No. U1240, Promega) and nuclease-free water in a 25 μl reaction volume. PCR analysis was carried out using 1 μl of 1:5 diluted cDNA in a reaction mixture containing 5 μl of Fast SYBR green 2× Master mix (Cat No. 4385612, Applied Biosystems, Life technologies), 0.5 μl each of 10 μm forward and reverse primer and the total volume adjusted to 10 μl using RNase-free water. The reaction was carried out in Applied Biosystems 7900HT Fast Real-Time PCR machine. The primer sequences are listed in Table 2.

**Table 1:** List of top 50 differentially expressed miRNAs in activated rat primary microglia.

No	miRNA	logFC	Fold change	*p*-value
1	rno-miR-29b-3p	1.79	3.45	1.54E-04
2	rno-miR-142-3p	2.43	5.37	6.76E-04
3	rno-miR-142-5p	1.80	3.49	4.08E-04
4	rno-miR-146a-5p	1.54	2.91	6.74E-03
5	rno-miR-101a-3p	1.36	2.57	2.20E-04
6	rno-miR-29c-3p	1.29	2.45	2.20E-04
7	rno-miR-146b-5p	1.26	2.39	1.31E-02
8	rno-miR-30e-5p	1.19	2.29	2.20E-04
9	rno-miR-9a-5p	1.17	2.25	3.07E-03
10	rno-miR-223-3p	1.16	2.23	4.73E-02
11	rno-miR-34a-5p	1.14	2.20	9.28E-04
12	rno-miR-101b-3p	1.11	2.16	2.86E-04
13	rno-miR-339-5p	1.08	2.12	4.08E-04
14	rno-miR-21-5p	1.07	2.10	4.08E-04
15	rno-miR-449a-5p	1.04	2.06	7.83E-03
16	rno-miR-19b-3p	1.00	2.01	4.68E-04
17	rno-miR-27a-3p	1.00	2.00	4.08E-04
18	rno-miR-370-5p	0.96	1.95	7.73E-04
19	rno-miR-9a-3p	0.93	1.90	4.55E-03
20	rno-miR-30a-5p	0.92	1.89	4.08E-04
21	rno-miR-19a-3p	0.92	1.89	4.66E-04
22	rno-miR-20b-5p	0.91	1.88	2.86E-04
23	rno-miR-20a-5p	0.91	1.88	1.25E-03
24	rno-miR-106b-5p	0.82	1.77	2.25E-03
25	rno-miR-24-2-5p	0.79	1.73	2.19E-03
26	rno-miR-33-5p	0.77	1.70	1.35E-02
27	rno-miR-29a-3p	0.76	1.70	2.19E-03
28	rno-miR-301a-3p	0.72	1.64	4.03E-03
29	rno-miR-17-5p	0.69	1.61	2.19E-03
30	rno-miR-30b-5p	0.69	1.61	2.75E-03
31	rno-miR-16-5p	0.65	1.57	2.25E-03
32	rno-miR-140-5p	0.64	1.56	3.04E-03
33	rno-miR-338-3p	0.64	1.56	1.25E-02
34	rno-miR-21-3p	0.63	1.55	1.51E-02
35	rno-miR-190a-5p	0.63	1.54	8.39E-03
36	rno-miR-27b-3p	0.60	1.52	6.19E-03
37	rno-let-7i-5p	0.59	1.51	1.81E-03
38	rno-miR-34b-5p	0.57	1.48	2.89E-02
39	rno-miR-29a-5p	0.55	1.47	1.65E-03
40	rno-miR-30d-5p	0.54	1.45	8.39E-03
41	rno-miR-30c-5p	0.51	1.42	1.66E-02
42	rno-miR-195-5p	0.50	1.41	7.37E-03
43	rno-miR-26b-5p	0.49	1.41	6.39E-03
44	rno-miR-9b-5p	0.49	1.41	3.78E-02
45	rno-miR-34c-5p	0.49	1.40	8.83E-03
46	rno-miR-148b-3p	0.40	1.32	1.00E-02
47	rno-miR-22-3p	0.37	1.29	3.86E-02
48	rno-miR-350	0.37	1.29	1.40E-02
49	rno-miR-872-5p	0.35	1.28	3.84E-02
50	rno-miR-107-3p	0.30	1.23	3.85E-02

**Table 2:** Primer Sequences.

Gene	Forward sequence (5′–3′)	Reverse sequence (5′–3′)
*Camk2a*	ATGCTGCTCTTTCTCACGCT	TTGTTTCCTCCGCTCTTCCC
*β-actin*	GGATTCCATACCCAAGAAGGA	GAAGAGCTATGAGCTGCCTGA

### miRNA Target Prediction

MiRNA microarray data were analyzed through the use of QIAGEN’s Ingenuity^®^Pathway Analysis (IPA^®^, QIAGEN Redwood City)^1^. The TargetScan website was used to confirm the miRNA: mRNA interactions in the 3′UTR of *Camk2a*.

### miRNA Knockdown and Overexpression

For functional analysis of miR-142-3p and miR-142-5p in microglia, BV2 cells were seeded in 6-well plates at a density of 2 × 10^5^. The gain of function studies were carried out using *mir*VANA^TM^ miRNA mimics (Cat. No. 4464066, 4464084, Life technologies) and negative control (Cat. No. 4464058, Life technologies). Loss of function studies were performed using *mir*VANA^TM^ microRNA inhibitors (Cat. No. 4464084, Life Technologies) and negative control miRNA (Cat. No. 4464076, Life Technologies). Transfection complexes were prepared in Opti-MEM medium (Cat. No. 31985070, Invitrogen, Life technologies) using X-tremeGENE siRNA transfection reagent (Cat. No. 04476093001, Roche Applied Sciences) following manufacturer’s instructions and were added to the cells at a final concentration of 20 nM, 30 nM, and 40 nM for mimics and 50 nM for inhibitors.

### Luciferase Assay

Luciferase assay was performed to verify if miR-142-3p targets the *Camk2a* 3′UTR. BV2 microglial cells were plated at a density of 2 × 10^5^ cells in 24-well plates. The luciferase vector containing the 3′TR of mouse *Camk2a* (NM_001286809.1) was commercially purchased from GeneCopoeia (Product ID: MmiT078538-MT06). Cells were co-transfected with mimics and negative control (40 nm) and luciferase vector (1,000 ng) using Lipofectamine^®^ RNAiMAX (Cat. No. 13778030, Thermo Fisher Scientific). The cells were cultured for 48 h after which luciferase activity was assayed according to the manufacturer’s instructions using Luc-Pair Duo-Luciferase Assay Kit 2.0 (Cat. No. LF001, GeneCopoeia). The luminescence intensity was measured using a luminometer (Spectramax M5) and firefly luciferase activity was normalized to renilla luciferase activity.

### Protein Extraction

For protein extraction from BV2 cells, about 2 × 10^5^ cells were seeded. The total protein was extracted from BV2 cells using the M-PER reagent (M-PER, Cat. No. 78501, Thermo Fisher) following the manufacturer’s protocol. The extracted protein was quantified using the Bradford method (Cat. No. 500-0006, Bio-Rad).

### Western Blotting

Thirty microgram of total protein from each sample was denatured at 95°C for 5 min and separated on a 10% SDS-PAGE. The proteins were transferred to polyvinylidene (PVDF) transfer membranes, blocked with 3% BSA and incubated with the primary antibodies, anti-CAMK2A antibody (rabbit polyclonal antibody, 1:1,000, Cat. No. A14012, Invitrogen), anti-BDNF antibody (rabbit recombinant monoclonal antibody, 1:1,000, Cat. No. ab108319, Abcam), anti-CREB antibody (rabbit monoclonal antibody, 1:1, 000; Cat. No. 9197, Cell Signaling Technology, Danvers, MA, USA) and anti-pCREB antibody [(pSer-133) rabbit monoclonal antibody, 1:1,000; Cat. No. 9198, Cell Signaling Technology, Danvers, MA, USA] overnight at 4°C. Following washing, blots were incubated with secondary Ms-HRP antibody (1:10,000, Cat. No. 31430, Thermo Fisher Scientific) or Rb-HRP antibody (1:10,000, Cat. No. 31460, Thermo Fisher Scientific) for 1 h at room temperature with gentle shaking. All blots were developed with enhanced chemiluminescence reagent (Clarity Western ECL Substrate, Cat. No. 1705060, Bio-Rad) and quantified on densitometer using Quantity One software (Bio-Rad). To normalize the protein content of each lane, the blots were stripped (Restore^TM^ PLUS Western Blot Stripping Buffer, Cat. No. 46430, Thermo Fisher Scientific) and re-probed with anti-beta actin (1:5,000, Cat. No. A2228, Sigma Aldrich) for total protein.

### Immunocytochemistry

Forty-thousand–60,000 BV2 microglial cells were seeded on poly-lysine coated coverslips in 24-well culture plates. Following transfection and LPS treatment, the cells were fixed with 4% PF, washed and blocked with 5% goat serum followed by incubation with the following antibodies: anti-CAMK2A antibody (mouse monoclonal antibody, 1:200, Cat. No. MA1-048, Thermo Fisher Scientific) and anti-BDNF antibody (rabbit recombinant monoclonal antibody, 1:200, Cat. No. ab108319, Abcam) overnight at 4°C. The cells were then incubated with secondary Rb-Cy3 antibody (1:200, Cat. No. C2306, Sigma-Aldrich) or Ms-Cy3 (1:200, Cat. No. C2181, Sigma-Aldrich) and lectin, a microglia specific marker (1:200, L0401, Sigma-Aldrich), followed by counterstaining with DAPI. The coverslips then mounted with fluorescent mounting medium (DakoCytomation, Glostrup, Denmark). Slides were allowed to dry for at least 1 day before imaging. Images were taken using LSM FV1000 (Olympus).

### Generation of CAMK2A Knockdown Stable Cells in Microglia

Stable knockdown of CAMK2A was performed by lentiviral mediated transduction of CAMK2A specific shRNA in BV2 microglial cells. Microglial cells were transduced with five shRNA individual clones (Dharmacon, GE Healthcare) against the *Camk2a* gene (Accession Number: NM_177407). Selection pressure was applied for 48 h after transduction using puromycin at the concentration of 2 μg/ml. Cells were maintained in puromycin containing medium for 6–10 days and expanded. The efficiency of knockdown in microglia was confirmed using western blotting analysis and the shCAMK2A clone that induced maximal knockdown was selected for further analysis.

### Statistical Analysis

Data is represented as mean ± SD from at least three independent experiments. Statistical significance was evaluated by either the Student’s *t*-test or one-way ANOVA analysis of variance followed by *post hoc* Tukey test. The data were considered significant at *p* < 0.05.

## Results

### Global miRNA Expression Profile Shows Differential Expression of miRNAs in Activated Microglia

**Figure 1:**
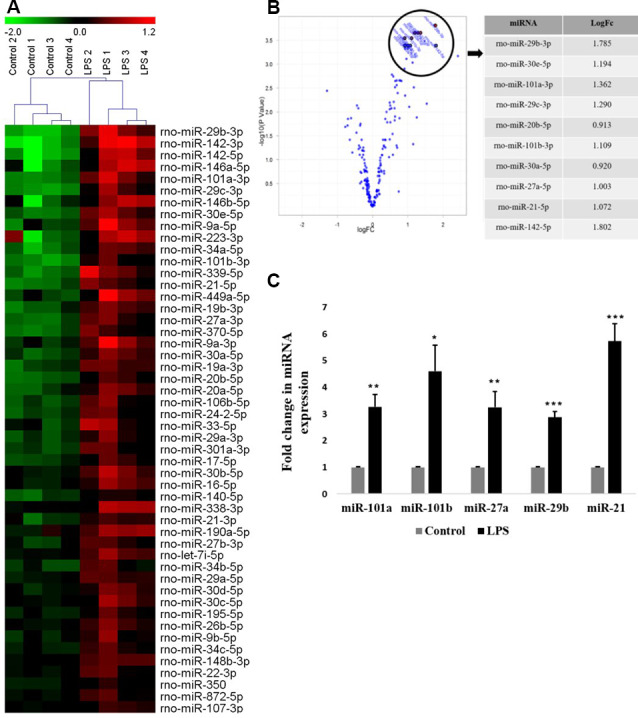
Global miRNA expression profiling shows the differential expression of miRNAs in activated microglia. **(A)** Heat map generated from microRNA microarray shows the result of differential miRNAs expression in control vs. lipopolysaccharide (LPS)-treated primary microglia. Each column represents a biological sample while each row represents a miRNA. The color scale represents the relative expression level of each miRNA in each sample as compared to the reference channel. Red color indicates an expression that is higher than the reference channel, while green color indicates an expression that is below the reference. **(B)** Volcano plot illustrating the relation between the logarithm of the *p*-values and the log fold change of miRNAs differentially expressed in control and LPS-activated microglia. The top selected miRNAs are marked with annotation on the plot and tabulated on the right. **(C)** Quantitative RT-PCR analysis of few differentially expressed miRNAs found in miRNA microarray in Control vs. LPS activated primary microglia. Results are represented as fold change for untreated controls. Statistical analysis was carried out using ANOVA with *post hoc* Tukey test. Data represented as mean ± SD, (*n* = 5), ***p < 0.001; **p < 0.01; *p < 0.05.

In the present study, the primary culture of microglia was prepared from the 3-day old rat pups cortices and grouped into two: untreated (control) and treated with LPS (activated). A global miRNA expression profiling of activated microglia was carried out using a microRNA microarray platform consisting of 3,100 unique capture probes (Exiqon miRCURY LNA^™^). We have identified and clustered the top 50 differentially expressed miRNAs in activated rat primary microglia compared to control, after stringent selection criteria applied (Figure 1A, Table 1). From the Principle Component Analysis (PCA) plot generated using the top 50 miRNAs, the biological difference between the microRNA expression profiles of activated and control microglia was very evident as shown by the separation of samples in different regions of PCA plot (Supplementary Figure S1). A volcano plot comparing the expression profile of control and LPS activated microglia identified members of miRNA-29, -30, -101, and -142 families as the most differentially expressed (Figure 1B).

To validate the miRNA profiling, the expression levels of top differentially expressed miRNAs such as miR-101a, miR-101b, miR-27a, miR-29b, and miR-21 were further confirmed in primary microglia by quantitative RT-PCR (Figure 1C) and were found to agree with the microarray data.

### Pathway Analysis of Differentially Expressed miRNAs in Activated Microglia Compared to Control Microglial Cells

To identify the potential signaling pathways affected by differentially expressed miRNAs in activated microglia and their target genes, pathway analysis was performed using IPA software. The pathway analysis revealed that the top two networks which include connective tissue disorders, inflammatory disease, and inflammatory response were the highest-rated network with 30 molecules involved. The highly affected molecular and cellular functions predicted by pathway analysis included: cell cycle, cellular development, cell death and survival, cellular growth and proliferation, and cell morphology (Supplementary Figure S2).

**Figure 2:**
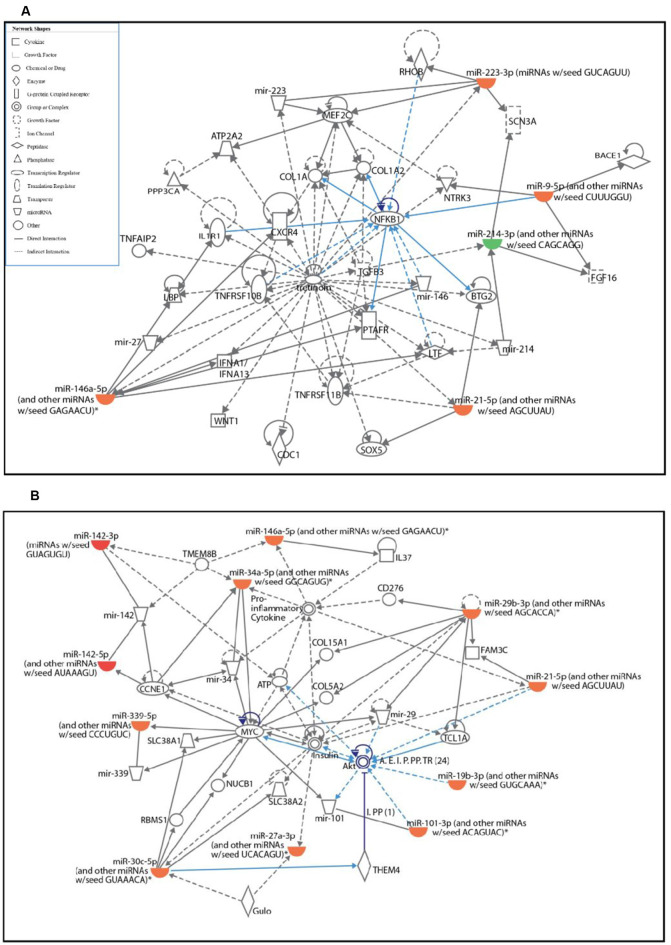
Ingenuity pathway analysis (IPA) analysis of differentially expressed miRNAs in activated primary microglia compared to control microglial cells. **(A,B)** IPA showing up- (red) and down-regulation (green) of miRNAs and their target gene networks such as NF-κB **(A)** and Akt **(B)** in LPS-activated primary microglial cells when compared to control.

The two significantly affected pathways in primary microglia cells upon activation are nuclear factor κ-light-chain-enhancer of activated B cells (NF-κB) signaling pathway (Figure 2A) and phosphatidylinositol 3-kinase (PI3K)/protein kinase B pathway (Figure 2B). Upon activation, the NF-κB signaling pathway in microglia is known to stimulate the transcription of pro-inflammatory genes coding for cytokines TNFα, IL-1β and IL-6, iNOS, and proteolytic enzymes inducing classical M1 pro-inflammatory phenotype (Kaminska et al., 2016). The role of PI3K/Akt pathway is also well-established in the microglial-mediated release of proinflammatory cytokines and inflammatory mediators *via* toll-like receptors, TLR4 upon stimulation by LPS (Medzhitov and Janeway, 2000; Oshiumi et al., 2008; Saponaro et al., 2012).

### miR-142-3p/5p Expression Is Upregulated in Activated BV2 Microglia

**Figure 3:**
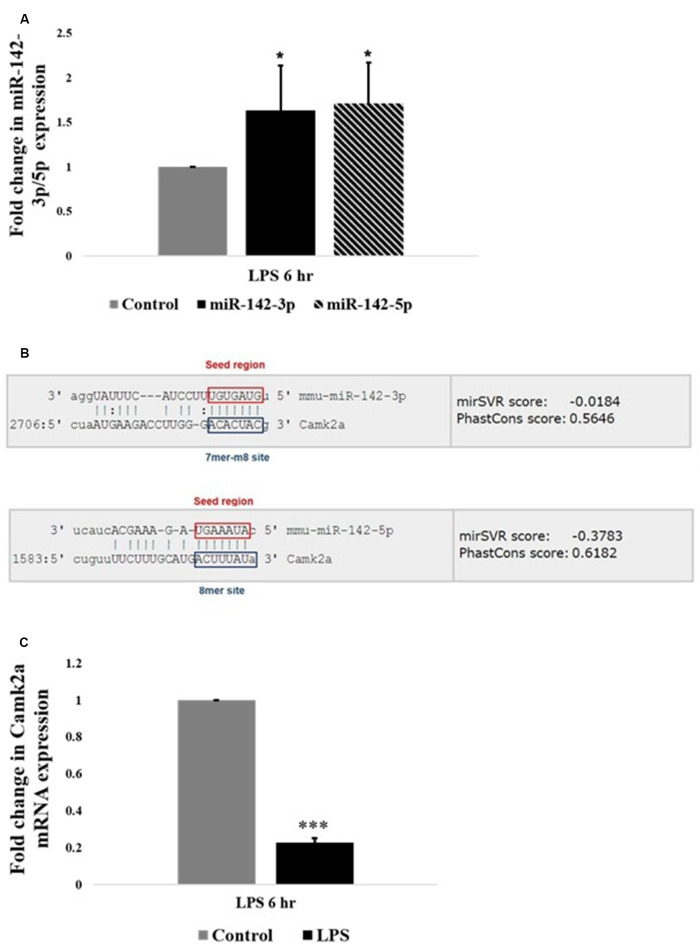
miR-142-3p/5p upregulates in activated microglia and targets CAMK2A. **(A)** Histogram depicts a significant upregulation of miR-142-3p and miR-142-5p in activated microglia upon LPS treatment for 6 h. Data represented as mean ± SD, (*n* = 5), Students *t*-test, *p < 0.05. **(B)** Bioinformatics analysis predicted that miR-142-3p and miR-142-5p putatively target 3′UTR of *Camk2a*. The seed region and the target site type have been highlighted (TargetScan and Miranda software was used to predict miRNA targets. Image acquired from http://www.microrna.org/). **(C)** Histogram depicting a significant decrease in mRNA expression levels of *Camk2a* in activated microglia upon LPS treatment for 6 h, suggesting an inverse relationship between miR-142-3p/5p and its target gene, *Camk2a*. Data represented as mean ± SD, (*n* = 4), Students *t*-test, ***p < 0.001.

Interestingly, among the highly upregulated microRNAs in activated microglia, miR-142 family is the most highly differentially expressed miRNA based on the fold change values. Further, quantitative RT-PCR analysis confirmed that the expression levels of miR-142-3p and miR-142-5p were upregulated in LPS activated BV2 microglia, compared to control untreated microglial cells (Figure 3A). MicroRNA target prediction analysis using TargetScan algorithms^2^ and Miranda^3^ software revealed that miR-142-3p and miR-142-5p putatively targets the 3′UTR region of *Camk2a* (Figure 3B). Given the recent finding where microglial BDNF, a neurotrophic growth factor known to be regulated by CaM kinases, was shown to promote learning-dependent synapse formation, we further investigated the role of miR-142-3p/5p mediated regulation of microglial BDNF through CAMK2A.

### Inverse Relationship Between miR-142-3p/5p and Its Target, *Camk2a*, Which Has a Putative Role in Synaptic Plasticity

CAMK2A is a subunit of CAMK2 protein, which is a ubiquitous serine/threonine-protein kinase present in the brain playing vital roles in synaptic plasticity, learning, and memory (Stephenson et al., 2017). The relation of miR-142-3p/5p and its target gene *Camk2a* was further pursued to understand the epigenetic regulation of microglial BDNF through CAMK2A under physiological and pathophysiological conditions. Quantitative RT-PCR analysis revealed an upregulation of miR-142-3p/5p expression in activated BV2 microglia. Concomitantly, a significant decrease in *Camk2a* mRNA expression was observed in activated BV2 microglia at 6 h of LPS treatment, indicating an inverse relationship between miR142-3p/5p and expression of its putative target gene, *Camk2a* in activated microglia (Figure 3C).

### CAMK2A-CREB-BDNF Pathway Is Downregulated in Activated Microglia

**Figure 4:**
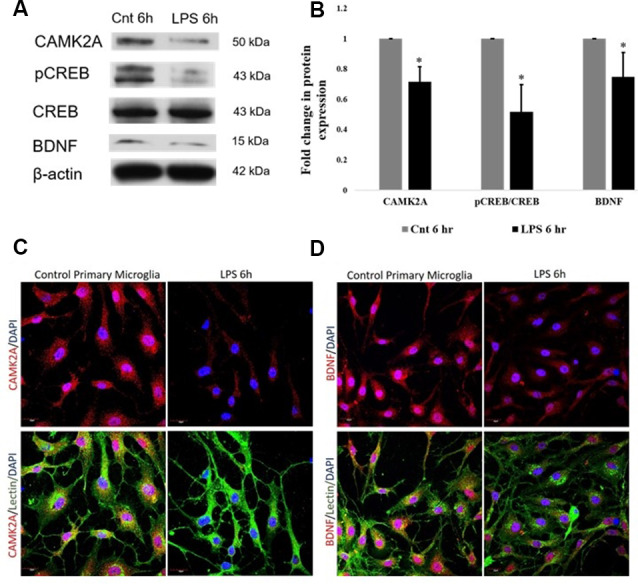
Downregulation of CAMK2A-BDNF pathway proteins in LPS-activated primary microglia. **(A)** Immunoblots of CAMK2A (50 kDa), phosphorylation of CREB (pCREB; 43 kDa), BDNF (15 kDa) in LPS activated primary microglia show significant downregulation. Total cyclic AMP-responsive element-binding protein (CREB) and β-actin show no difference between control and activated microglia. **(B)** Histogram shows the significant downregulation of CAMK2A, pCREB, and BDNF in microglia treated with LPS for 6 h. Immunoblot data has been quantified and normalized to β-actin. Phospho-CREB was then normalized to total CREB. Data represented as mean ± SD, (*n* = 3), Students *t*-test, *p < 0.05. **(C)** Immunofluorescence analysis shows that CAMK2A (red) expression appears to be decreased in primary microglia treated with LPS for 6 h when compared to controls. **(D)** Immunofluorescence analysis shows that BDNF (red) expression also appears to be decreased in primary microglia treated with LPS for 6 h when compared to controls. Lectin (green) used as microglial markers. Nuclei are stained with DAPI (blue), (*n* = 3), Scale bars = 20 μm.

The protein expression of CAMK2A in activated primary rat microglia was evaluated. CAMK2A protein was found to be significantly decreased in primary microglia activated by LPS treatment for 6 h (Figures 4A,B). A critical function of CAMK2A is the phosphorylation of CREB, a transcription factor that mediates the transcription of BDNF (Yan et al., 2016). Western blot analysis revealed a significant decrease in pCREB and BDNF expression level in activated microglia following LPS treatment for 6 h (Figures 4A,B). These results were further verified using immunofluorescence, wherein, a stark decrease in CAMK2A expression was noticed in activated microglia when compared to control (Figure 4C). Further, immunofluorescence analysis revealed a decrease in BDNF expression in microglia following LPS treatment (Figure 4D). These results indicate that the CAMK2A-CREB-BDNF pathway is downregulated in activated microglia.

### miR-142-3p Regulates CAMK2A in Microglia

**Figure 5:**
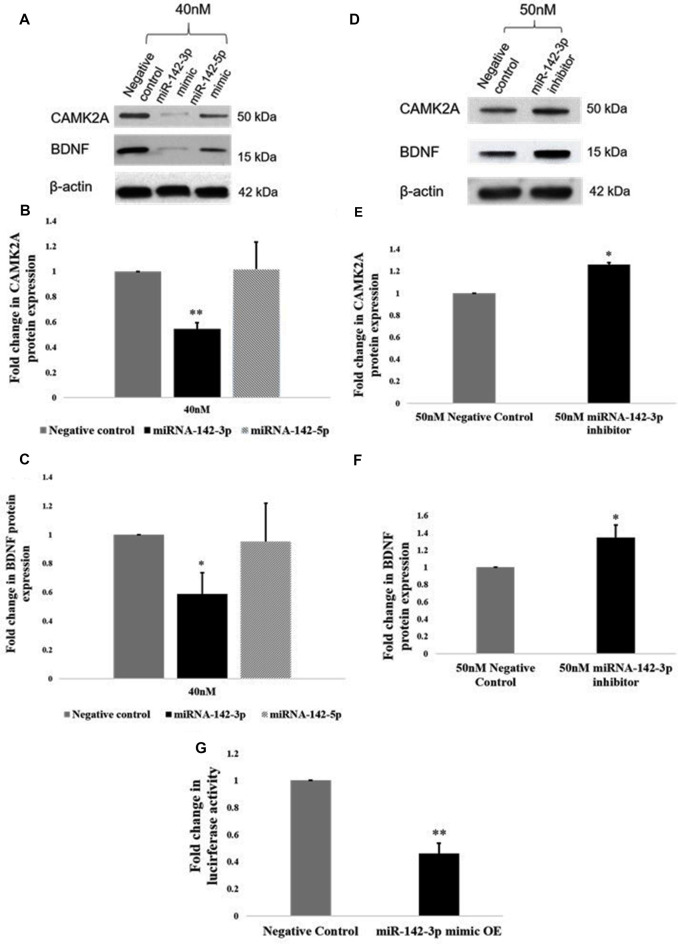
miR-142-3p targets CAMK2A and regulates the CAMK2A-BDNF pathway in microglia. **(A–C)** Immunoblot and densitometry analysis shows that overexpression of mir-142-3p mimics suppresses the protein expression levels of CAMK2A and BDNF in BV2 microglia significantly. However, overexpression of miR-142-5p did not suppress CAMK2A protein expression levels. Data represented as mean ± SD, (*n* = 3), Students *t*-test, **p < 0.01; *p < 0.05. **(D–F)** Immunoblot and densitometry quantification shows that overexpression of mir-142-3p inhibitors in BV2 microglia leads to an increase in the protein expression levels of CAMK2A and BDNF, suggesting an inverse relationship between miR-142-3p and CAMK2A. Immunoblot data has been quantified and normalized to β-actin. Data represented as mean ± SD, (*n* = 3), Students *t*-test, *p < 0.05. **(G)** The histogram shows a significant decrease in the luciferase activity in BV2 cells co-transfected with miR-142-3p mimics and luciferase vector as compared to cells co-transfected with negative control miRNA and luciferase vector, indicating that miR-142-3p targets CAMK2A. Data represented as mean ± SD, (*n* = 3), Students *t*-test, **p < 0.01.

Given that an inverse relationship between miR-142-3p/5p and CAMK2A has been established in activated microglia, we sought to establish a functional relationship. Functional analysis of miR-142-3p/5p in microglia was carried out using miR-142-3p/5p mimics and inhibitors to simulate the overexpression and knockdown of miR-142-3p/5p in BV2 microglia, respectively. To determine the transfection efficiency of the miRNA mimics, BV2 cells were transfected at different concentrations of 20 nM, 30 nM, and 40 nM, where 40 nM yielded the prominent effect at the protein expression. Overexpression of miR-142-3p led to a significant decrease in the protein expression of CAMK2A as compared to negative control cells (transfected with scrambled miRNAs, however overexpression of miR-142-5p did not significantly alter CAMK2A expression in microglia (Figures 5A,B). These results suggested that miR-142-3p effectively targets CAMK2A in microglia. Hence, miR-142-3p was chosen for further studies. Next, BV2 microglia were transfected with miR-142-3p inhibitors and negative control miRNAs at 40 nM and 50 nM concentrations where 50 nM yielded the prominent effect. MiR-142-3p inhibitors transfection in BV2 microglia revealed a significant increase in CAMK2A protein levels when compared to control (Figures 5D,E).

Further, a 3′UTR luciferase assay was performed to confirm that *Camk2a* is a target of miR-142-3p. BV2 microglial cells were transfected with a luciferase vector containing the 3′UTR of the mouse *Camk2a* gene together with miR-142-3p overexpression (mimics) or negative control miRNAs. A significant decrease in the luciferase activity in BV2 microglia was observed upon co-transfection of the mimics and the luciferase vector, indicating that the miR-142-3p binds to the 3′UTR of *Camk2a* (Figure 5G). Taken together, these results confirm that CAMK2A is a target of miR-142-3p in microglia.

### miR-142-3p Mediated Downregulation of CAMK2A Leads to Repression of BDNF, a Learning and Memory Molecule

Next, we determined the effect of miR-142-3p on the expression of BDNF. Upon overexpression of miR-142-3p, we observed a ~50% significant decrease in protein levels of BDNF (Figures 5A,C), and conversely, inhibition of miR-142-3p resulted in a significant increase in the expression of BDNF (Figures 5D,F). Altogether, these findings indicate that miR-142-3p epigenetically regulates the expression levels of BDNF through CAMK2A in activated microglia.

### shRNA Mediated Knockdown of CAMK2A Suppresses the Expression of BDNF *via* CREB

Given the promiscuous nature of miRNA-mediated gene regulation, it is important to ascertain if the regulation of BDNF expression by miR-142-3p occurred through its target CAMK2A.

**Figure 6:**
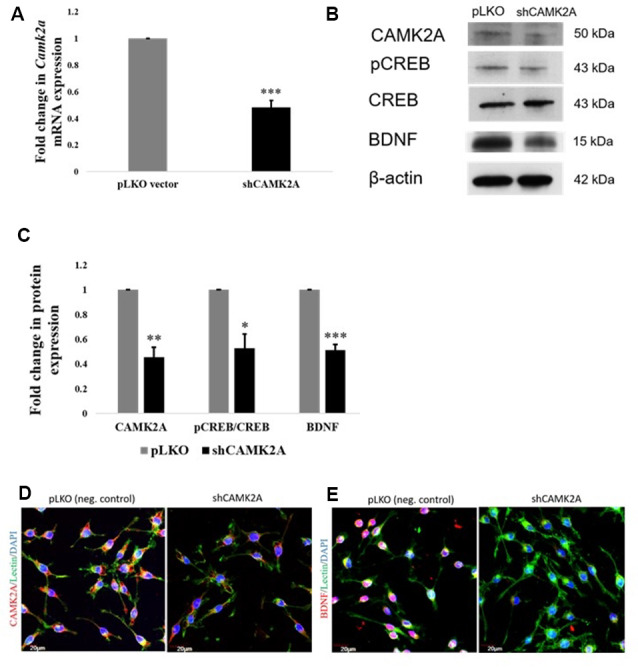
shRNA mediated knockdown of CAMK2A suppresses the CAMK2A-BDNF pathway in microglia. **(A)** Quantitative RT-PCR results show a significant decrease in the mRNA levels of *Camk2a* in BV2 microglia following the shRNA-mediated knockdown of CAMK2A (shCAMK2A). Data represented as mean ± SD, (*n* = 4), Students *t*-test, ***p < 0.001. **(B)** Immunoblot shows that the shRNA mediated knockdown of CAMK2A leads to the downregulation of BDNF *via the* downregulation of pCREB in BV2 microglia when compared to negative control plasmid (pLKO) transfected microglial cells. **(C)** The histogram shows the significant downregulation of CAMK2A, pCREB, and BDNF in shCAMK2A microglia, compared to negative control plasmids (pLKO) transfected microglial cells. Immunoblot data has been quantified and normalized to β-actin. Phospho-CREB was then normalized to total CREB. Data represented as mean ± SD, (*n* = 3), Students *t*-test, ***p < 0.001; **p < 0.01; *p < 0.05. **(D)** Immunofluorescence analysis shows that CAMK2A (red) expression appears to be decreased in shCAMK2A microglial cells compared to negative control plasmids (pLKO) transfected microglial cells. **(E)** Immunofluorescence analysis shows that BDNF (red) expression appears to be decreased in shCAMK2A microglial cells compared to negative control plasmids (pLKO) transfected microglial cells. Lectin (green) used as microglial markers. Nuclei are stained with DAPI (blue), (*n* = 3), Scale bars = 20 μm.

To achieve this, a stable knockdown of CAMK2A in microglia was generated by the transduction of small-hairpin RNA (shRNA) against the *Camk2a* gene. shRNA-mediated knockdown of the *Camk2a* gene resulted in a ~50% decrease in mRNA levels of *Camk2a* (Figure 6A) as well as protein levels of CAMK2A (Figures 6B,C). Western blot analysis in stable knockdown cells confirmed the regulation of BDNF expression by CAMK2A through phosphorylated CREB in comparison to negative control which is an empty vector (pLKO) transduced BV2 cells (Figures 6B,C). A consistent downregulation of CAMK2A, pCREB, and BDNF at the protein level was observed in microglia after knockdown of CAMK2A compared to a negative control. This observation was further confirmed by immunocytochemistry showing a decrease in the expression level of CAMK2A and BDNF in shRNA-mediated CAMK2A knockdown cells compared to the empty vector transduced cells (Figures 6D,E). Overall, these results demonstrate that miR-142-3p-mediated downregulation of CAMK2A in microglia leads to the repression of BDNF, which is involved in synaptic plasticity.

## Discussion

Microglia protect the brain parenchyma against various insults *via* several effector functions such as secretion of proinflammatory cytokines, chemokines, and cytotoxic factors as well as phagocytosis of cellular debris (Thameem Dheen et al., 2007). On the other hand, microglia derived factors such as BDNF, have been shown to contribute to the maintenance of synaptic plasticity (Harada et al., 2002; Parkhurst et al., 2013). Thus, any disruption of microglial functions in the brain could affect microglial-mediated neuroinflammation and synaptic integrity (Hoshiko et al., 2012; Schafer et al., 2012). It has recently been established that neuroinflammation in the brain could negatively impact memory and cognitive functions, as a result of altered expression of memory-associated genes (Baghel et al., 2018). Given that microglial activation is a hallmark of neuroinflammation, there is an urgent need for understanding the molecular and epigenetic mechanisms that underpin microglial functions in the healthy and pathological brain. Recently, miRNAs targeting several genes are involved in regulating microglia behavior and functions in diverse CNS pathologies including ischemic brain injuries (Selvamani et al., 2012; Zhao et al., 2013; Karthikeyan et al., 2018), multiple sclerosis (Ponomarev et al., 2011) and prion disease (Saba et al., 2012; Slota and Booth, 2019). In the present study, miRNA profiling of activated microglial cells provided a comprehensive catalog of differentially expressed miRNAs in activated microglia, and the pathway analysis identified altered expression of several miRNAs targeting genes involved in neuroinflammation as well as synaptic plasticity in activated microglia. For example, some of the differentially expressed miRNAs such as miR-21 and the miR-29 family in the activated microglia were found to target genes involved in microglia-mediated neuroinflammation (Zhang et al., 2012; Thounaojam et al., 2014). Several other miRNAs such as miR-101, miR-142-3p, which were among the top hits obtained in the miRNA microarray in the present study, are expressed in peripheral macrophages, but their functions in microglia remain unknown (Zhu et al., 2010; Xie et al., 2014).

The pathway analysis further revealed that the NF-κB signaling pathway which plays an important role in neuroinflammation was upregulated in activated microglia (Dheen et al., 2005; Shih et al., 2015). Specifically, this analysis showed upregulation of miR-146a-5p, miR-95p, miR-223-3p, and miR-21-5p in activated microglia. These microRNAs were found to regulate several genes such as BTG2 (B-cell translocation gene 2), which is induced in response to LPS in macrophages in NF-κB-mediated manner (Kawakubo et al., 2004; Witham et al., 2013), RhoB (Ras Homolog Family Member B), a known target of miR223-3p (Fritz and Kaina, 2001; Sun et al., 2009) that has been reported to repress NF-κB in murine fibroblasts cells (Fritz and Kaina, 2001; Rodriguez et al., 2007), C-X-C Motif Chemokine Receptor 4 (CXCR4), which is involved in inducing IL-6 and chemokine production through activation of NF-κB signaling pathway (Lu et al., 2009) and IL1R1 (Interleukin 1 Receptor Type 1) which is involved in cytokine-induced activation of NF-κB signaling (Andreakos et al., 2004).

Pathway analysis further revealed that several miRNAs including miR-29b-3p, miR-21-5p, miR-19b-3p, miR-101-3p, and miR-30c-5p, were found to be upregulated in activated microglia. These miRNAs target different members of the PI3K/Akt (protein kinase B) pathway, which is involved in the NF-κB-dependent inflammatory response of activated microglia (Saponaro et al., 2012) and long-term potentiation (LTP) through BDNF (Saw et al., 2020).

While there is an adequate understanding of miRNA-mediated regulation of the inflammatory response of microglia during pathology, there is a surprising dearth of information on the miRNAs that regulate microglia-mediated synaptic maintenance. In the present study, miR-142-3p which was found to be upregulated in activated primary microglia was shown to target CAMK2A, which is a multifunctional protein kinase known to be critical for the execution of cellular Ca^2+^ signal transductions and functions as a molecular substrate for LTP, forming the molecular basis of learning and memory (Liu and Murray, 2012).

Furthermore, *loss-of-function* and *gain-of-function* studies confirmed that miR-142-3p targets CAMK2A affecting the CAMK2A-CREB-BDNF signaling pathway in microglia. It has been shown that CAMK2A-mediated pCREB is involved in Ca^2+^ induced expression of BDNF which is imperative for neuronal signaling and neurite outgrowth (Yan et al., 2016). Here, for the first time, we have shown that the miR-142-3p negatively regulates the CAMK2A-CREB-BDNF signaling pathway in activated microglia. The understanding of the epigenetic regulation of BDNF in microglia is crucial as BDNF has been shown to activate neuronal Tropomyosin-related kinase receptor B (TrkB) that regulates synaptic plasticity and modulate glutamatergic synaptic transmission *in vivo* and neuronal LTP, which is a key feature of memory formation and consolidation (Parkhurst et al., 2013; Lai et al., 2018).

In this study, we demonstrated that BDNF expression is regulated by miR-142-3p, the highly upregulated miRNA upon microglial activation, through its target CAMK2A. Several studies have shown that miR-142-3p functions as a regulator of neuroinflammation by modulating the expression of proinflammatory mediators in response to different pathological stimuli, such as multiple sclerosis, neuropathic pain, human immunodeficiency virus (HIV) and simian immunodeficiency virus encephalitis (SIVE; Chaudhuri et al., 2013; Talebi et al., 2017; Zhang et al., 2018). This study reveals a novel role of miR-142-3p in activated microglia as its upregulation upon microglial activation perturbs the CAMK2A-CREB-BDNF signaling pathway, which is involved in synaptic plasticity. It has been previously reported that activated microglial cells in septic animals exhibit heightened secretion of pro-inflammatory factors that result in synaptic impairment, which forms the cellular basis of memory and cognitive decline (Moraes et al., 2015). Moreover, microglial activation coupled with the secretion of pro-inflammatory factors in the aging brain has been shown to depress LTP (Griffin et al., 2006). Thus, miR-142-3p mediated regulation of microglia-derived neurotrophic and pro-inflammatory factors in neuropathologies may form a basis for deficits in synaptic maintenance and memory and cognitive decline.

As inhibitors of microglial activation have been shown to reverse cognitive deficits and neuroinflammation, the identification of miR-142-3p as a regulator of microglia-derived BDNF may aid in the development of new therapeutic strategies that assist in rescuing cognitive functions during the neuropathological condition.

**Figure 7:**
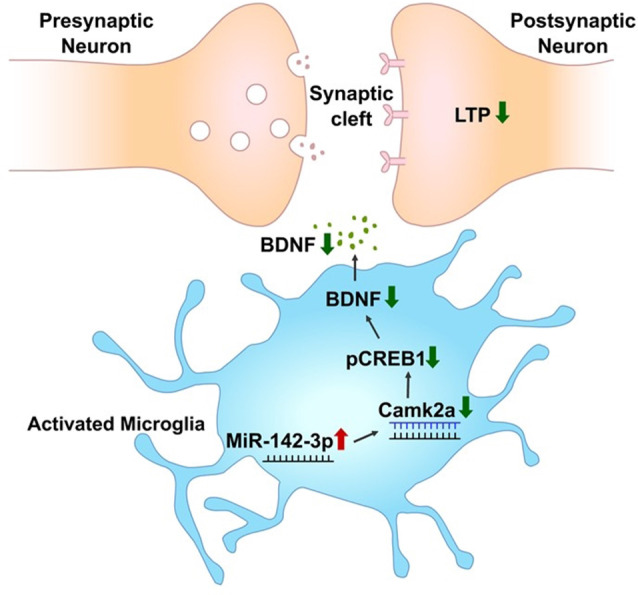
miR-142-3p epigenetically regulates the CAMK2A-BDNF pathway in microglia. In the activated microglia, miR-142-3p was upregulated, leading to suppression of its target, CAMK2A protein expression, which eventually downregulates the level of BDNF through decreased pCREB (red arrow indicates upregulation and green arrow indicates downregulation). This illustration suggests that under pathological conditions, the CAMK2A-BDNF pathway which is known to be involved in learning and memory is epigenetically regulated in activated microglia.

In summary, this study provides a database of differentially expressed miRNAs in activated primary microglia. Through this screening, miR-142-3p targeting CAMK2A which is involved in learning and memory through the CREB-BDNF signaling pathway was found to be upregulated in activated microglia (Figure 7).

## Data Availability Statement

The raw data supporting the conclusions of this article will be made available by the authors, without undue reservation, to any qualified researcher.

## Ethics Statement

The animal study was reviewed and approved by the Institutional Animal Care and Use Committee of the National University of Singapore under the IACUC protocol no. R14-1643. All procedures were under IACUC guidelines.

## Author Contributions

NG, SJ, K-LT, GS, and SD: manuscript conception and design. SJ and NG: performed microRNA microarray, data acquisition, and analysis. NG: validation of the CAMK2A-BDNF pathway and wrote the manuscript. KM: generated shRNA-mediated CAMK2A knockdown cell lines. SD: provided intellectual contribution, edited the manuscript, and is the principal investigator of the study.

## Conflict of Interest

The authors declare that the research was conducted in the absence of any commercial or financial relationships that could be construed as a potential conflict of interest.

## Footnotes

^1^http://www.qiagen.com/ingenuity

^2^http://www.targetscan.org/mmu_71/

^3^http://www.microrna.org/
