# Supplementary figures and images for: miR-142-3p Regulates BDNF Expression in Activated Rodent Microglia Through Its Target CAMK2A
Source: Front Cell Neurosci. 2020 May 21;14:132. doi: 10.3389/fncel.2020.00132 (PMC7253665; doi:10.3389/fncel.2020.00132)

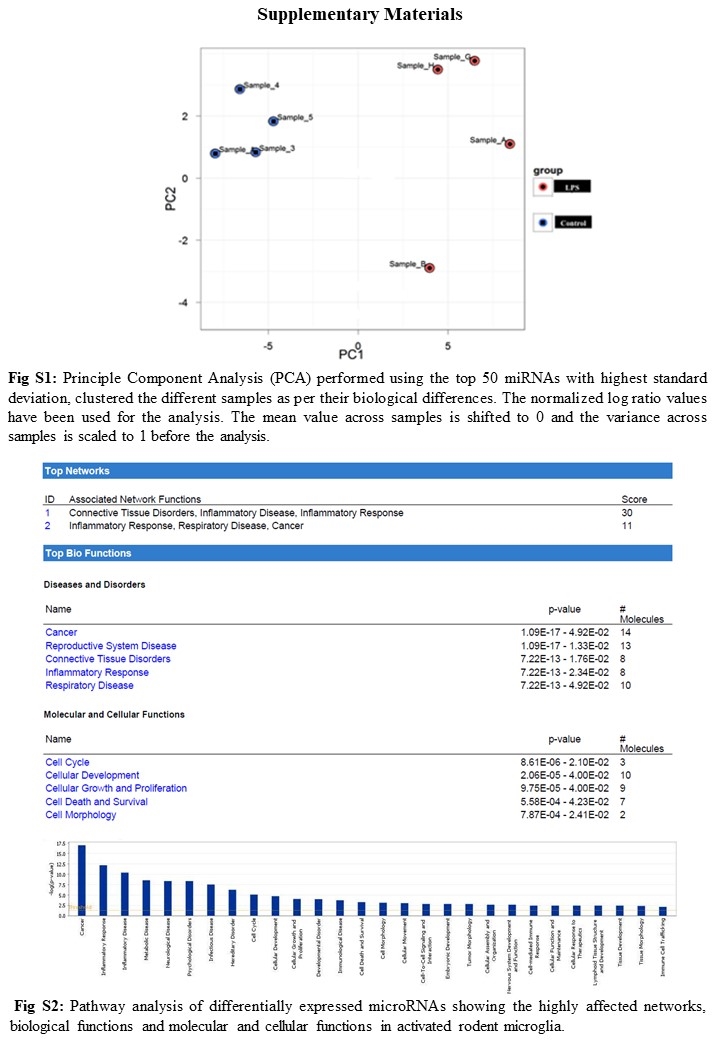

Supplement: Supplementary file 1 [file Image_1.JPEG]

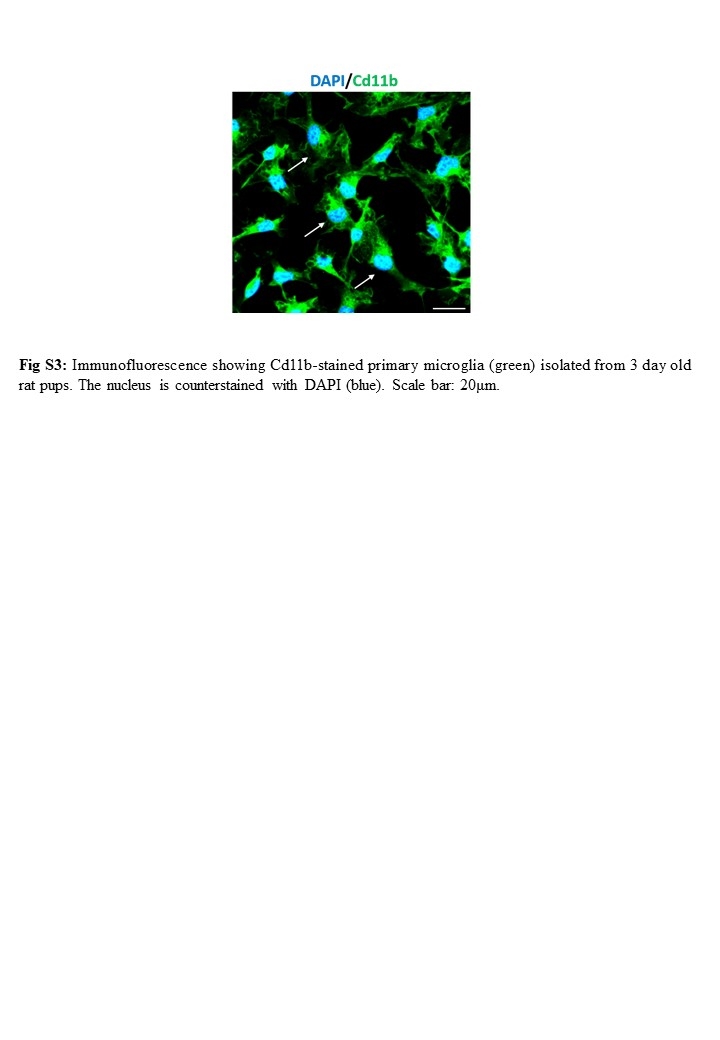

Supplement: Supplementary file 2 [file Image_2.JPEG]
